# Characteristics and Challenges of Primary Adrenal Insufficiency in Africa: A Review of the Literature

**DOI:** 10.1155/2022/8907864

**Published:** 2022-08-24

**Authors:** Thabiso R. P. Mofokeng, Salem A. Beshyah, Ian L. Ross

**Affiliations:** ^1^Division of Endocrinology, Department of Medicine, University of the Free State, Bloemfontein, South Africa; ^2^Dubai Medical College for Girls, Dubai, UAE; ^3^Yas Clinic Group Hospital, Khalifa City, Abu Dhabi, UAE; ^4^Division of Endocrinology, Department of Medicine, University of Cape Town, Cape Town, South Africa

## Abstract

**Backgrounds:**

Africa comprises 54 countries with varying degrees of economic development. As with other healthcare systems, rare diseases such as adrenal insufficiency are neglected and poorly documented.

**Objectives:**

We wished to explore primary adrenal insufficiency (PAI) in Africa, its prevalence, aetiology, genetics, presentation, diagnosis, and treatment and to determine the unmet needs in clinical care, education, and research.

**Materials and Methods:**

A narrative nonsystematic review of the literature was undertaken. We searched two online databases (PubMed and Google scholar) using the search terms “Addison's disease/PAI, primary adrenal insufficiency coupled with “Africa,” “country names,” and “genetic disorders.” A total of 184 PAI records were reviewed. The exclusion of abstracts, conference proceedings, single case reports, and duplicate studies covering the same subject matter yielded 124 articles, of which 97 informed the final manuscript.

**Results:**

A wide range of aetiology of PAI was encountered, but their true prevalence is unknown. Aetiology varied with region and age of presentation as reflected by predominantly TB, HIV, and infective causes occurring in sub-Saharan Africa and more congenital forms described in North Africa associated with consanguinity. In Africa, the diagnostic criteria for PAI do not universally accord with conventional criteria, and there is a heavy reliance on clinical suspicion and biochemistry, including random cortisol of <400 nmol/L, rather than the tetracosactide test where stimulated cortisol <500–550 nmol/L confirms the diagnosis.

**Conclusions:**

A high index of suspicion is warranted to diagnose PAI in resource-limited settings, especially where tetracosactide tests are not universally available.

## 1. Introduction

Primary adrenal insufficiency (PAI) is a life-threatening disorder due to glucocorticoids' deficient production and function. The most common causes of PAI in developed countries are autoimmune, infectious aetiology, bilateral adrenal haemorrhage, bilateral infiltration, adrenal metastases, drugs, and adrenalectomy [1]. Availability of diagnosis and treatment of this disease remains challenging, especially in low and middle-income countries (LMIC) in Africa, with profound healthcare resource limitations [2].

Africa comprises 54 countries of varying degrees of economic development [3]. As with many healthcare systems, in predominantly under-resourced systems, rare and neglected diseases are poorly documented relative to prevalence and morbidity [4]. Several critical challenges face healthcare systems in Africa. For example, Oleribe et al. [5] identified the main problems as inadequate human resources, poor health resource allocation, deficient health system maintenance, and lack of political will. Malakoane et al. [6] found fragmentation of the health services, staff shortages, poor governance, and financial resource limitations to be the main problems in the Free State of South Africa, a relatively large geographical region.

Patients with PAI present with nonspecific, insidious symptoms of cortisol deficiency, including fatigue, weakness, listlessness, orthostatic dizziness, weight loss, and anorexia. Some patients initially present with gastrointestinal symptoms, such as abdominal cramps, nausea, vomiting, and diarrhoea, and in other patients, the disease may be misdiagnosed as depression or anorexia nervosa [7]. Difficulty in diagnosing PAI among black Africans may, among other things, be due to hyperpigmentation being less discernible [8] and its symptoms being falsely attributable to more prevalent communicable diseases such as HIV and *tuberculosis*.

There is a noticeable dearth of information on PAI in Africa despite the presumed enormous burden of infectious diseases. Large-scale studies evaluating the true prevalence of PAI have not been undertaken [9]. We hypothesized that there may be unmet needs in diagnosis, lack of uniformity in respect of diagnosis, clinical care, and research. To test this hypothesis, we reviewed the literature on PAI in Africa, aiming to ascertain its prevalence, aetiology, genetics, presentation, diagnosis, and treatment. Highlighting these aspects of PAI should help improve the outcome of this eminently treatable condition in this specific geographical region.

## 2. Materials and Methods

**Figure 1:**
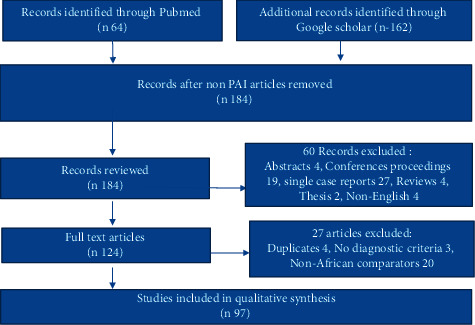
Flowchart of the article screening and selection process.

A review of the available literature was undertaken. We searched PubMed and Google Scholar online databases and used the search terms “Addison's” disease or PAI in conjunction with the search term “Africa,” the “individual country name,” and “genetic disorders.” No date limitations were introduced, and a total of 184 PAI records were reviewed. The exclusion of abstracts, conference proceedings, single case reports, and duplicate studies covering the same subject matter yielded 124 articles, of which 97 informed the final manuscript (Figure 1). Articles of the crucial importance of non-African origin were reviewed to compare to other regions or inform some arguments.

## 3. Results

### 3.1. Epidemiology of Adrenal Insufficiency

Relative to the size of the African continent and its population, there are very few publications describing the aetiology of PAI. Few patients are identified in most African hospitals failing to keep records of rare conditions. Hence, there are significant limitations in drawing robust conclusions about a particular disorder [10]. In an extensive study of PAI performed in South Africa, most of the patients were of European descent rather than black Africans, who comprise the most significant proportion of the population [11].

A single case-finding study of PAI in South Africa documented 3.1 cases per million, considerably fewer than in Western countries [12]. Possible reasons for the lower prevalence include difficulty diagnosing PAI among black Africans due to less discernible hyperpigmentation and the occurrence of autoimmunity which may be lower in Africa than in Western countries. On the other hand, the low ascertainment of autoimmunity may be due to under testing. The high background population of HIV and *tuberculosis* in Africa may also contribute to this underdiagnosis, as symptoms of PAI may be misconstrued as being associated with these aforementioned communicable diseases [12]. In a survey of physicians managing patients with PAI in South Africa, though not designed to determine the prevalence, we inferred a prevalence of 14.2 per million [13]. This prevalence is much lower than the reported 144 per million in Western countries [14].

### 3.2. Aetiology of Adrenal Insufficiency in Africa

Descriptions of PAI in Africa are limited to case reports and only occasional extensive cohort studies, in contrast to Western countries, where extensive cohort studies chronicle in detail most aspects of this condition, including drugs such as mitotane and etomidate, genetic conditions, adrenal cancer metastases, infections (bacterial, viral, and fungal), adrenal haemorrhage, diminishing incidence of tuberculosis, and increasing autoimmune aetiology [15–30].

#### 3.2.1. Autoimmune PAI

In a large study of PAI in South Africans, the majority of whom were of European descent and females, [11] with over half of the 144 studied patients deemed to have autoimmunity as the underlying cause based on the presence of either positive 21-hydroxylase autoantibodies or adrenocortical autoantibodies [11]. This is at variance from Western countries, where most PAI is due to autoimmunity. It may, in part, be explained by the long interval between diagnosis and screening for antibodies, with a median of 12 years and a range of 50 years, respectively. The actual proportion of autoimmunity in the South African cohort might have been more significant if the autoantibodies were sampled soon after the diagnosis of AI. The prevalence of 21-hydroxylase autoantibodies supports this in 90% of patients with autoimmune PAI of recent onset disease (<2 years) and only 79% in those longer than two years duration [31]. Thus, the lower-than-expected proportion with autoimmune PAI may be due to the gradual waning of antibodies over time and thus may have been underestimated [32].

#### 3.2.2. Autoimmune PAI Association with Autoimmune Polyglandular Syndrome APS 1 & 2

In cases of autoimmune PAI, either in association with autoimmune polyglandular syndrome 2 (APS 2) or as isolated autoimmune PAI in Western countries, the human leukocyte antigen (HLA) region has strong associations [14]. In a South African study, 5 (3%) patients had APS 1 and 66 (46%) had APS 2 [11]. Interestingly, despite a substantial geographic distinction, a comparison of autoimmune PAI cohorts of South African and American populations revealed similar high-risk haplotypes in patients with 21-hydroxylase autoantibodies, namely (DRB1^*∗*^0301-DQB1^*∗*^0201 (DR3) and DRB1^*∗*^04xx-DQB1^*∗*^0302 (DR4)). The presence of these haplotypes in geographical regions with unlikely common ancestry indicates the relative importance of these high-risk HLA alleles for developing autoimmune PAI [33]. Fourati et al. [34] described non-HLA autoimmunity-derived APS 2 in 60 Tunisian patients, 40 of whom had incomplete APS 2 and 20 had complete APS 2, indicating the coexistence of PAI in the latter cohort, without traditional HLA high-risk haplotypes.

#### 3.2.3. Congenital Causes of Adrenal Insufficiency Disease

In contrast to Western countries where there are studies documenting PAI resulting from autoimmunity, genetic disorders, and infective aetiology [35], there are sparse data concerning the aetiology of PAI in Africa. The genes that are involved in the aetiology of PAI in the Western world have been described by Betterle et al. [36], whereas only a subset of these has been described in Africa (Supplementary Material S1).

*(1) Allgrove Syndrome*. Triple-A (Allgrove) syndrome is a rare, adrenocorticotrophic (ACTH)-resistant, autosomal recessive hereditary disease, caused by mutations of the triple-A (AAAS) gene, which encodes for the protein ALADIN (alacrima achalasia, adrenal insufficiency, and neurologic disorder), located on chromosome 12q13. Mutations of this gene have been reported in consanguineous North African and European families [37]. Tebaibia et al. [38] reported a higher prevalence of Allgrove syndrome among PAI cohorts of 7.7% in an Algerian versus 1.0% in a South African study by Ross et al. [39], respectively. Consanguineous marriages are more common in North Africa than in sub-Saharan Africa, ranging in incidence from 20% in Morocco to more than 30% of all marriages in Tunisia [40, 41]. Berrani et al. [42] described a Moroccan brother and sister born to consanguineous parents who had the (c.1331 + 1G > A) mutation of the AAAS gene, which is one of the most commonly described genes in North African countries, for example, Tunisia, Algeria, and Libya.

*(2) Familial Glucocorticoid Deficiency*. Familial glucocorticoid deficiency (FGD) is a rare, autosomal recessive disorder characterised by mutations of the ACTH receptor (melanocortin two receptor, MC2R) or the melanocortin two receptor accessory protein (MRAP), which is required for trafficking MC2R to the cell membrane. These mutations cause FGD types 1 and 2, respectively, resulting in resistance to the action of ACTH, leading to the adrenal cortex's failure of glucocorticoid production [43]. Affected individuals are deficient in cortisol, with preserved mineralocorticoid production, and if untreated, are likely to succumb to hypoglycaemia or overwhelming infection in infancy or childhood [44]. These mutations account for 45% [45] MC2R (25%) and MRAP (20%), and steroidogenic acute regulatory protein (STAR) account for (5%) of FGD [46]. The median age at presentation is variable, ranging from 0.02 years to 16 years, and the children commonly present with hypoglycaemia and hypoglycaemic convulsions. Tall stature is associated with mutations in MC2R but not in MRAP. FGD type1 presents later in life, in contrast with FGD type 2, presenting earlier with normal adult height [44, 47, 48].

Patients with FGD are usually diagnosed in the neonatal period or early childhood. The diagnosis is based on clinical findings of hyperpigmentation, jaundice, hypoglycaemia, seizures, failure to thrive, and frequent and intercurrent severe infections [48]. Our search revealed single case reports of this condition mainly concentrated in North African countries such as Morocco, Algeria, Tunisia, Egypt, Ethiopia, and Sudan. More recently, novel nicotinamide nucleotide transhydrogenase [49] or thioredoxin reductase 2 (TRXR2) mutations have been found [50]. Nicotinamide nucleotide transhydrogenase [49] is a highly conserved gene coding for a mitochondrial protein protecting cells from oxidative stress. Novoselova et al. [51] described a novel heterozygous variant (p.Arg71^*∗*^) in NNT, which was inherited in two affected siblings with an East Asian mother and a South African father who presented with increased skin and gum hyperpigmentation. Roucher-Boulez et al. [52] analysed the NNT gene in 50 patients with PAI, and 7 new NNT mutations were reported, including p.Met337Val, p.Ala863Glu, c.3G > A (p.Met1), p.Arg129^*∗*^, p.Arg379^*∗*^, p.Val665Profs^*∗*^29, and p.Ala704Serfs^*∗*^19. The most frequent mutation p.Arg129^*∗*^ was found repeatedly in patients from Algeria with a history of consanguinity.

Steroidogenic acute regulatory protein (StAR) deficiency is a rare but important cause of PAI. This gene mutation causes severe steroid hormone deficiency, resulting in hypoglycaemia, salt loss, adrenal hyperplasia, and adrenal lipoid deposition [53]. In the African setting, FGD reports are mainly single case and conference reports. Achermann et al. reported 2 Libyan sisters with a history of consanguinity, who presented with biochemically proven severe PAI in infancy and radiologically enlarged adrenals containing lipoid deposits. They were found to have lipoid congenital adrenal hyperplasia (CAH) due to a novel homozygous T to G transversion within the splice donor site of exon one intervening sequence (IVS1 + 2T > G, g.66). Both patients responded adequately to glucocorticoid and mineralocorticoid therapy [54].

*(3) Congenital Adrenal Hyperplasia*. Congenital adrenal hyperplasia (CAH) is most frequently caused by 21-hydroxylase deﬁciency (21-OHD), which is inherited in an autosomal recessive pattern. This condition is classified as a classical phenotype, salt-wasting (SW), simple virilising (SV) form, and nonclassical (NCAH) form, as manifested by androgen excess such as acne, premature pubic hair, advanced bone age, and reduced adult height. Dehydration associated with an SW syndrome and ambiguous genitalia in the newborn should suggest CAH. The classical form occurs from 1 in 10 000 to 1 in 15 000 in the general population, whereas the NCAH form is more common, occurring in 1 in 1 000 people [55].

In Egypt, Elmougy et al. [56] screened 174 unrelated children with 21-hydroxylase-deficient CAH for genetic mutations, with the majority of the cohort (45.9%) having either I2 splice or p.Q318X deletions, whereas 7.4% of the cases were negative for all mutations. The most infrequently detected mutations were p.P453S, cluster E6.pR483P, and p.L307FS, occurring in 5% of the cohort. In Tunisia, Charfeddine et al. [57] determined the genetic defect CYP21A2 in 50 Tunisian patients with the clinical diagnosis of 21-hydroxylase deficiency, with CYP21A2 mutations being identified in 87% of the alleles, whereas the most common point mutation was the pseudogene specific variant p.Q318X (26%). In a retrospective study on 43 patients with a disorder of sexual development (DSD) and 38 with adrenal insufficiency, Niang et al. [58] reported that 32 had CAH, representing 74.4% and 84.2% of the causes of DSD and adrenal insufficiency, respectively. Of these 32, 27 (84.4%) were girls and 5(15.6%) were boys. Due to lack of neonatal screening, the diagnosis of CAH was delayed, leading to life-threatening adrenal crises in 75% of females with observed genital ambiguity, in whom adrenal insufficiency was missed at birth. In South Africa, Ganie et al. [59] described 44 children with classic CAH, with the majority (59.8%) having manifested with classic salt-wasting (CSW) CAH and 40.1% having had the simple virilising (SV) form. Two-thirds of females with 46XX CAH and all males with 46XY CAH presented with dehydration and shock, resulting from an SW syndrome in this study of South African patients. In the paediatric age group, CAH should be included in the differential diagnosis of hypoglycaemia and vascular collapse in infants [60].

*(4) Zellweger Syndrome*. Zellweger syndrome (ZS) is a peroxisome biogenesis disorder attributed to a mutation of the 13 peroxins (PEX) genes, with an incidence estimated at 1 in 50 000 in North America [61]. The incidence of this disease in Africa and the Arab world remains unknown. Peroxisomal enzyme deficiencies accumulate very-long-chain fatty acids (VLCFAs) but lack plasmalogens [62]. Plasmalogens are unusual phospholipids, which form essential components of various membranes and structures of high-fat content, peak in myelin, and red blood cells. Their function remains unknown in specific membranes, particularly those of the nervous system, immune, and cardiovascular cells, where they are highly abundant [63]. Cardinal manifestations of ZS [61] include low muscle tone, facial dysmorphism, impaired growth, sensory and neurological dysfunction, renal and endocrine insufficiency, skeletal abnormalities, and developmental delay.

Data suggest predominance in the North rather than sub-Saharan Africa. Of a total number of 52 patients, with ZS confirmed on the VLCFA positive test, Nasrallah et al. [62] in Tunisia reported only 4 cases of PAI as a result of ZS manifesting with elevated ACTH and low cortisol concentrations. All patients exhibited neurological impairment at birth, dysmorphia, and polymalformations in 65% of patients younger than three months of age. There were no positive predictive factors for the development of adrenal insufficiency in the four patients who were diagnosed with PA [62].

*(5) Adrenoleukodystrophy*. Adrenoleukodystrophy (ALD) is a rare, X-linked disorder of peroxisomal oxidation, but it is the most common peroxisomal disease due to mutations in the ABCD1 gene. It leads to reductions in ABCD1 (adrenoleukodystrophy protein) and downstream to very high concentrations of very-long-chain fatty acids (VLCFAs) in plasma, which accumulate in the white matter of the brain spinal cord and the adrenal cortex. It manifests with PAI, myelopathy, and cerebral involvement. PAI occurs in over 80% of ALD patients [64]. This condition penetrates 100% in males and 65% in heterozygous females by 60 years. In our South African cohort of 147 patients with Addison's disease, 4% had X-linked adrenoleukodystrophy, based on elevated very-long-chain fatty acids, without genetic analyses. Nevertheless, our Africa-wide survey suggested that it occurred among 2.6% of the total cohort of Addison's disease in Africa, indicating some consistency in the actual proportion of the cohort and one reported in our survey [9].

#### 3.2.4. Infective Causes of Adrenal Insufficiency

*(1) Tuberculosis*. For more than a century, the adrenal glands have been the most commonly involved endocrine organs in *tuberculosis* [65]. Relative to other causes of PAI in Africa, this has been described in greater detail for this continent than for other continents, possibly due to the high *tuberculosis* burden in Africa [66, 67]. The frequency of adrenal insufficiency among patients with pulmonary *tuberculosis* TB in sub-Saharan Africa is reported to range between 0.9% and 59% [68], depending on the region where it is endemic. In the oldest report of an infective cause of PAI in Africa, Taube reported two symptomatic PAI cases, occurring from *tuberculosis* in Zimbabwe in 1970 [69]. In a retrospective study of 50 patients with acute PAI, presenting to a South African teaching hospital from 1980 to 1997, the underlying aetiology was active *tuberculosis* in 18%. In another study, previous *tuberculosis* based on positive chest radiology or calcified adrenal glands on either computed tomography (CT) or plain abdominal films was also detected in 16% [70]. In our recent Africa and the Middle East survey, *tuberculosis* was a more frequent cause of PAI in sub-Saharan Africa (34%) than in the MENA region^99999^ (4.1%) [9]. Also, *tuberculosis* as a cause of PAI was more often diagnosed in South African public health services than in the private sector (42% vs. 6%; *p* < 0.001. Nevertheless, the data are limited by recall bias in our survey [9]. Studies by Odeniyi in Nigeria, Mugusi in Tanzania, and Soule in South Africa suggest that *tuberculosis* is still an important cause of PAI with reported 14%, 30%, and 18%, respectively (Table 1). This African prevalence rate is similar to the one reported by Nerup [71] in 1974 in Denmark, where 17 of 108 PAI patients were reported to be due to *tuberculosis*. However, in a more recent study of PAI by Betterle et al. [16] in Italy, in contrast to Africa, of 633 patients with PAI, 57 (9%) were as a result of *tuberculosis*, 29 (4.6%) had genetic conditions, 10 (1.6%) had adrenal cancer, 4 (0.6%) had vascular disorders, and 32 (5.1%) were defined as idiopathic, emphasizing the differences in Africa and Europe in terms of the relative contributions of infective causes.

*(2) Human Immunodeficiency Virus (HIV)*. Adrenal insufficiency due to *tuberculosis* and HIV appears to be particularly well described in SSA. For example, in a cross-sectional study of 66 hospitalised HIV patients in a semiurban setting in South Africa, the prevalence of hypoadrenalism, defined as exhibiting a basal cortisol concentration below 400 nmol/L, was 27%. Each of the patients identified as hypoadrenal also had cytomegalovirus, a marker of severe immune paresis, and 62% had concurrent *tuberculosis* [72]. In a retrospective study in Ghana, Sarfo-Kantanka et al. [73] reported 29 patients with PAI based on short tetracosactide tests, albeit their diagnosis threshold was not reported. Of these patients, 14 had HIV-associated adrenalitis and two had disseminated *tuberculosis*.

#### 3.2.5. Sickle Cell Disease-Associated Adrenal Insufficiency

Sickle cell disease (SCD) is common in West Africa, with estimates that it affects 230 000 children in SSA per year [74]. It has been associated with PAI development, utilising the tetracosactide test as a diagnostic modality. Endocrine dysfunctions are known to occur in sickle cell anaemia. This impairment is suggested to occur due to recurrent occlusion of small vessels and as an outcome of oxidative stress-mediated organ toxicity led by iron overload [75]. Sobngwi et al. [75] in Cameroon investigated 10 SCD patients and ten controls who underwent a 250 *μ*g tetracosactide test, which recorded poststimulation incremental cortisol of 132.8 nmol/L versus 207.36 nmol/L, *p*=0.047, respectively. According to their predetermined criteria, 50% of their SCD patients failed to reach optimal cortisol responses, indicating that patients with SCD are likely to have a high burden of primary hypoadrenalism, but the studies are limited by their small sample sizes.

### 3.3. Dermocorticoid Use

In sub-Saharan Africa, voluntary, medically unsupervised depigmentation using dermocorticoids is widespread, with the prevalence ranging from 27% to 67% according to Hode et al. [76] Although the focus of our review is PAI and its management, we felt that it is necessary to highlight this widespread practice, which can potentially lead to the suppression of the entire HPA axis consistent with tertiary hypoadrenalism and fatal consequences.

### 3.4. Clinical and Biochemical Characteristics of AI in Africa

The cardinal clinical features elicited from the cohort of patients with PAI, who presented on average 12 years previously, were hyperpigmentation in 76%, nausea and vomiting in more than 40%, and weight loss in 25%. As a presenting feature, loss of consciousness occurred in 20%, suggesting a substantial delay in the diagnosis and a significant proportion who presented with an acute adrenal crisis [77]. The veracity of these data may be limited by recall bias. In South Africa, Soule et al. [70] reported in the cohort of acute PAI that patients presenting to a tertiary hospital had typical symptoms of confusion and loss of consciousness, which suggest a late presentation. The possibility exists that people may be dying before this diagnosis could be made and appropriate treatment instituted. In Western countries, the presentation appears to be less dramatic, with hyperpigmentation as the most distinctive feature and symptoms including loss of appetite, unintentional weight loss, nausea, and abdominal and muscle pain. Symptoms may progress over months and culminate in an adrenal crisis [78] if left untreated.

To make a diagnosis of PAI, Husebye et al. [78] recommended a stimulated serum cortisol concentration of less than 412 nmol/L at 30 minutes and less than 485 nmol/L at 60 minutes using liquid chromatography-tandem mass spectrometry, following the 250 *μ*g tetracosactide test. They report that many people who do not reach the threshold lower limit of normal at 30 minutes may do so at 60 minutes; hence, the recommendations of double sampling are made to avoid overdiagnosis of PAI. Other authors have suggested relying on immunoassays to indicate PAI, to use a baseline ACTH twice the upper limit of normal, with an increment of cortisol of less than 200 nmol/L and a peak cortisol level less than 500 nmol/L in response to the ACTH stimulation [79].

The tetracosactide test is not readily available in Africa, including South Africa, where it is necessarily acquired with permission from the South African Health Products Regulator (SAHPRA). As a result, several studies in Africa have applied different diagnostic criteria and different tetracosactide-stimulated cortisol results from those described in Europe to diagnose PAI. In South Africa, Ekpebegh et al. [72], for example, utilised a basal cortisol concentration of less than 400 nmol/L, with or without symptoms of PAI. In comparison, Mpe et al. [80] used random or stimulated cortisol of less than 550 nmol/L to diagnose adrenal insufficiency in intensive care unit (ICU) patients. This cortisol cut-point was also used by Ross et al. [39] In Sudan, Elhassan et al. [81] used a random cortisol cutoff of less than 500 nmol/L, and Nasralla et al. [62] in Tunisia used an elevated plasma ACTH and low serum cortisol without providing specific cutoffs. It has been proposed from European data that random cortisol of 200 nmol/L, in combination with elevated ACTH, should be used to diagnose PAI with high specificity and sensitivity when the tetracosactide test is not available [19].

### 3.5. Glucocorticoid Replacement And Optimization of Therapy

There is variable access to replacement therapy across 54 African countries, influenced by urbanisation and a difference in private versus public healthcare settings. Our Africa-wide survey [9] revealed the use of a wide variety of glucocorticoid replacement therapies, including hydrocortisone, prednisone, methylprednisolone, and dexamethasone, depending on availability with a greater range available in the private health system, compared to the public health system (44.0% vs. 17.2%; *p* < 0.001). In a survey examining the knowledge and attitude of patients with adrenal insufficiency, Bouziane et al. [82] found that 74% of the patients did not have sufficient understanding of their disease and the risks associated with it, including crises precipitated by stress and sickness requiring dose adjustments. In Tunisia, Chihaoui et al. [83] reported that adult Muslim populations in predominantly North African regions who uphold Ramadan have the attendant risk of potentially lethal hypoglycaemic events. Therefore, special precautions including risk stratification and closer monitoring of PAI patients are advised in order to minimize hypoglycaemic crises, which may be fatal, especially in the high-risk group.

The use of glucocorticoids in clinical medicine is ubiquitous, but their impact on cardiovascular and lipid and lipoprotein metabolism is not always appreciated during long-term treatment or replacement. The lipid and lipoprotein responses to glucocorticoids are highly variable due to the number of factors, which influence their metabolism, including doses, genetic variations, and environmental factors [84]. Ross et al. [84] found that South African patients with PAI have a more atherogenic profile, with elevated high-sensitivity C-reactive protein (hs-CRP), low-density lipoprotein cholesterol (LDL-C), triglycerides (TGs), and lower high-density lipoprotein cholesterol (HDL-C), compared with matched healthy controls (Table 2).

Patients with PAI also report impairment of attention and memory, even in replacement therapy, which was corroborated in a South African study of PAI, using the Brief Test of Adult Cognition by Telephone (BETACT), an objective measure of cognition [85]. It is evident from the self-reported survey that patients with PAI may experience disrupted sleep architecture, which is associated with the poor quality of life, leading to difficulty in consolidating memory [86]. Patients with PAI appear to show more significant impairment in verbal learning and memory loss than controls, likely attributable to their disordered sleep, characterised by poorer sleep efficiency and more time spent awake, and may be a function of their imperfect supplementation with glucocorticoids [87]. As patients with PAI are prone to poorer quality of life, depressed mood, and memory impairment, compared with healthy controls, using a latent variable model, it appears that disrupted sleep is responsible for inducing poorer quality of life, depressed mood, and impairment of memory, rather than PAI per se [88]. However, in their review paper, Esposito et al. [89] attributed reduced QOL in PAI patients to underuse of fludrocortisone.

## 4. Conclusions

PAI, which was once universally fatal, is now eminently treatable if diagnosed and treated promptly. PAI in Africa has many presentations and causes. Certain genetic defects, especially monogenic disorders, predominate among paediatric forms of PAI and geographical regions where consanguinity is prevalent. Autoimmunity in North Africa and South Africans of European descent appear to predominate, and a variety of infective causes, particularly *tuberculosis* in sub-Saharan Africa, have also been recognized. A clustering of the autoimmune polyglandular syndrome (APS) and adrenoleukodystrophy (ALD) in Morocco and South Africa was described. Also, Allgrove syndrome is seen more often in North Africa than in southern Africa. Across Africa, the diagnostic criteria for PAI vary and may not universally accord with conventional criteria. There is a heavy reliance on clinical suspicion and biochemistry including low random cortisol rather than dynamic testing.

Provision of qualified human resources and both reliable and readily available laboratory systems in Africa are vital for timely recognition and trustworthy confirmation of genetic disorders. The awareness among medical practitioners in Africa that *tuberculosis* and HIV infections may cause PAI is crucial given the observed excess relative frequency of these conditions in Africa. Continuous patient education is necessary to optimize compliance and improve care and QOL by adjusting therapy, especially during sick days and Ramadan fasting, to avoid adrenal crises.

## Figures and Tables

**Table 1:** Summary of available cohort studies investigating the prevalence and aetiology of primary adrenal insufficiency in Africa under various clinical scenarios.

Authors (year)	Number	Sex : F : M	Country	Findings and context	Ref
Ross et al. 2010	148	91 : 57	RSA	The cohort of PAI patients (based on 250 *μ*g tetracosactide and cortisol cutoff of <550 nmol/L associated with an elevated plasma ACTH (50%) had an autoimmune aetiology, and 65% of them were of European descent.	[11]
Ross et al. 2013	148	91 : 57	RSA	In the PAI cohort, in which the most common signs were hyperpigmentation (76%), nausea and vomiting in more than 40%.	[12]
Fourati et al. 2012	60	44 : 16	Tunisia	In APS patients, of whom 20 were found to have Addison's disease in varying association with other autoimmune conditions, e.g., T1D (73.3%), POF (3.3%), and AITD (91.7%).	[34]
Kallabi et al. 2016	26	14 : 12	Tunisia	A genetic study of Allgrove syndrome revealed a primary homozygous mutation (c.1331 + 1G > A) in 25 patients and R286X mutation in one patient. PAI was found in all patients on clinical grounds and an elevated plasma ACTH and reduced random cortisol concentrations.	[37]
Broodryk. 2010	73	49 : 24	RSA	Sputum-positive TB patients underwent the tetracosactide test; 5 had hypoadrenalism defined by serum cortisol <500 nmol/l	[67]
Odeniyi et al. 2013	43	20 : 23	Nigeria	HIV-positive patients underwent the tetracosactide test; 15 (34.8%) had hypoadrenalism defined as serum cortisol <380.2 nmol/l.	[90]
Odeniyi et al. 2017	44	No mention	Nigeria	Two groups underwent the tetracosactide test; one group had TB alone, whereas the other group had TB & HIV. 14 (31.1%) and 2 (5%) of the former and latter groups, respectively, had hypoadrenalism defined as cortisol <380.2 nmol/l.	[66]
Mugusi et al. 1990	50	No mention	Tanzania	Chronic TB patients underwent the tetracosactide test; 16 (32%) had hypoadrenalism by stimulated plasma cortisol <600 mmol/l.	[91]
Ekpebegh et al. 2011	66	39 : 27	RSA	HIV-positive patients underwent the tetracosactide test; hypoadrenalism (based on cortisol cutoff of <400 nmol/L) was diagnosed in 27%.	[72]
Musa et al. 2021	80	31 : 49	Sudan	422 suspected with suggestive symptoms were screened using the 250 mcg tetracosactide test, and 80 met the criteria. The morning cortisol level was <27.6 nmol/L in 70 and borderline 27.7 nmol/L–331.1 nmol/L in 10 patients with elevated ACTH levels. Aetiology ranged from Allgrove syndrome (29/80), APS (9/80), ALD (7/80), bilateral haemorrhage (1/80), and unspecified (34/80).	[81]
Odeniyi et al. 2011	44 : 70	21 : 23 35 : 35	Nigeria	44 sputum-positive TB patients and 70 controls underwent the tetracosactide test (hypoadrenalism defined as 30 minutes cortisol <380 nmol/L or increment from baseline of <158 nmol/L); of the 44 TB patients, 5 males and 5 females met the hypoadrenalism criteria.	[92]
Mabuza et al. 2020	75	32 : 43	RSA	TB patients underwent the 1 mcg tetracosactide test: 28 (37.3%) met the hypoadrenalism criteria of 30 minutes serum cortisol of <550 nmol/L.	[93]
Akase et al. 2019	350	178 : 172	Nigeria	HIV-positive patients on ART underwent the 1 mcg tetracosactide test: 57 (16.3%) met the 30 minutes criteria of serum cortisol <500 nmol/L.	[94]

PAI: primary adrenal insufficiency; APS: antiphospholipid syndrome; ALD: adrenoleukodystrophy; TB: *tuberculosis*; HIV: human immunodeficiency virus: ART: antiretroviral therapy; TID: type 1 diabetes; POF: premature ovarian failure; AITD: autoimmune thyroid disease; RSA: Republic of South Africa.

**Table 2:** A summary of cohort studies on the management and monitoring of adrenal insufficiency (PAI) reported from Africa.

Authors (year)	Sex F : M	Country	Findings and context	Ref
ILR 2013	31 : 30	RSA	Evaluation of salivary cortisol [57] concentrations in healthy controls versus PAI patients on hydrocortisone replacement therapy revealed greater concentrations of SF in PAI patients at all sampling points except at 08h00; however, there was no correlation between the hydrocortisone dose with the AUC for cortisol.	[77]
ILR 2016	25 : 26	RSA	SF and cortisone [57] measured in PAI patients and controls did not appear to be valuable biomarkers for monitoring hydrocortisone replacement in PAI as neither of these parameters correlated with a hydrocortisone dose.	[95]
ILR 2014	139 : 147	RSA	Examination of the relationship between metabolic parameters and 9*β* polymorphism did not reveal significant associations.	[96]
ILR 2014	55 : 55	RSA vs Sweden	The cardiovascular risk in the South African group versus the Swedish group revealed worse lipid profiles and higher hs-CRP levels in the RSA group, despite using lower doses of hydrocortisone in the Swedish group of PAI.	[97]

SF: salivary cortisol; SE: salivary cortisone; PAI: primary adrenal insufficiency; QOL: quality of life; AUC: area under the curve; hs-CRP: highly sensitive C-reactive protein; RSA: Republic of South Africa.

## Data Availability

All the primary studies from which the data were extracted are cited in the manuscript, and the extracted data are included within the manuscript.

## Supplementary Materials

Supplementary Materials Supplementary Material S1: summary of the genetic and neonatal etiologies of PAI which have been described in the Western world compared to the ones described in Africa. ()
