# Supplementary material for: Characteristics and Challenges of Primary Adrenal Insufficiency in Africa: A Review of the Literature
Source: Int J Endocrinol. 2022 Aug 24;2022:8907864. doi: 10.1155/2022/8907864 (PMC9433274; doi:10.1155/2022/8907864)
Supplement: Supplementary Materials — Supplementary Material S1: summary of the genetic and neonatal etiologies of PAI which have been described in the Western world compared to the ones described in Africa. () [file 8907864.f1.doc]

**Supplementary Material** **S1**:

| Genetic and neonatal causes of PAI which have been described in: | **The West** | **N-Africa** | **SSA** |
| --- | --- | --- | --- |
| **Impaired steroidogenesis with congenital adrenal hyperplasia (CAH)**  21-hydroxylase deficiency (CYP21A2 mutation)  11β-hydroxylase deficiency (CYP11B1 mutation)  3β-hydroxysteroid dehydrogenase type 2 deficiency (HSD3B2 mutation)  17α-hydroxylase deficiency (CYP17A1 mutation)  P450 oxidoreductase deficiency (POR mutation)  P450 side-chain cleavage deficiency (CYP11A1 mutation)  Aldosterone synthase deficiency (CYP11B2 mutation)  Cortisone reductase deficiency (HSD11B1 mutation)  Apparent cortisone reductase deficiency (H6PDH mutation | X  X  X  X  X  X  X  X  X | X  X  X  X  X | X  X |
| Congenital lipoid adrenal hyperplasia (CLAH | X | X | X |
| **Bilateral congenital adrenal hypoplasia or adrenal dysgenesis**  X-linked adrenal hypoplasia congenita (NR0B1/dosage-sensitive sex reversal X-linked gene 1 or DAX-1 mutation)  Steroidogenic factor 1 deficiency (NR5A1 encoding or SF-1 mutation)  IMAGe syndrome (CDKN1C encoding cyclin-dependent kinase inhibitor 1C gene mutation)  MIRAGE syndrome (SAMD9 mutation)  Pallister–Hall syndrome (GLI3 mutation)  Meckel syndrome (MKS1 mutation)  Pena–Shokeir syndrome (DOK7, RAPSN mutation)  Pseudotrisomy 13  Hydrolethalus syndrome (HYLS1 mutation)  Galloway–Mowat syndrome (WDR73 mutation) | X  X  X  X  X  X  X  X  X  X | X  X |  |
| **Familial glucocorticoid deficiency (FGD) and FGD-like conditions**  FGD type 1 (MC2R encoding the ACTH receptor or melanocortin 2 receptor mutation)  FGD type 2 (MRAP encoding the MC2R-ancessory protein responsible for translocation of the ACTH receptor to the membrane mutation)  FGD type 3 (StAR mutation, see CLAH)  FGD type 4 (NNT encoding nicotinamide nucleotide transhydrogenase mutation)  FGD-DNA repair defect (MCM4 mutation, natural killer cell and glucocorticoid deficiency with DNA repair defect) | X  X  X  X  X  X | X  X  X  X |  |
| **Congenital adrenal destruction (e.g., adrenoleukodystrophy, adrenomyeloneuropathy, adrenal calcification, adrenalitis)**  ABCD1 or ABCD2 genes encoding for a peroxisomal membrane transporter protein, X-linked  PEX 1 (neonatal adrenoleukodystrophy)  LIPA gene mutation (bilateral adrenal calcification)  AIRE gene mutation (lymphocyte autoimmune adrenalitis) | X  X  X  X | X  X  X |  |
| Kearns–Sayre syndrome (mitochondrial DNA deletions) | X |  | X |
| **Cholesterol synthesis disorders**  Wolman disease (LIPA mutations)  Smith–Lemli–Opitz syndrome (DHCR7 mutations)  Abeta-lipoproteinemia (MTP mutation)  Familial hypercholesterolemia (LDRL gene mutation)  Sitosterolemia (ABCG5 gene mutation) | X  X  X  X |  | X  X |
| **Metabolic lysosomal disorders**  Sphingosine-1-phosphate lyase 1 deficiency (SPGL1 gene mutation) | X |  |  |
| Triple A syndrome or Allgrove’s syndrome (Triple A gene AAAS encoding WD-repeat protein ALADIN) | X | X | X |
| Zellweger syndrome (PEX1 and other PEX genes mutation) | X | X | X |
| Infantil Refsum disease (PHYH and PEX7 genes mutations) | X |  |  |
| Maternal Cushing’s syndrome (transient adrenal deficiency due to foetal pituitary–adrenal axis suppression) | X |  |  |
| Autoimmune Polyglandular Syndrome type 1 (AIRE gene mutation) | X | X | X |
| North Africa (N-Africa), Sub-Saharan Africa (SSA)  Modified from Betterle et al.^37^ | | | |
